# False Positive 18F-FDG Uptake in Mediastinal Lymph Nodes Detected with Positron Emission Tomography in Breast Cancer: A Case Report

**DOI:** 10.1155/2013/459753

**Published:** 2013-03-04

**Authors:** Gamze Uğurluer, Mustafa Kibar, Sinan Yavuz, Akin Kuzucu, Meltem Serin

**Affiliations:** ^1^Department of Radiation Oncology, Acibadem Adana Hospital, Acibadem University School of Medicine, Seyhan, 01130 Adana, Turkey; ^2^Department of Nuclear Medicine, Acibadem Adana Hospital, Seyhan, 01130 Adana, Turkey; ^3^Department of Internal Medicine, Acibadem Adana Hospital, Acibadem University School of Medicine, Seyhan, 01130 Adana, Turkey; ^4^Department of Thoracic Surgery, Inonu University School of Medicine, 44000 Malatya, Turkey

## Abstract

Breast cancer is the most frequently diagnosed cancer among females. It is accepted that lymph node involvement with metastatic tumor and the presence of distant metastasis are the most important prognostic factors. Accurate staging is important in determining prognosis and appropriate treatment. Positron emission tomography with computed tomography detects malignancies using 2-[18F]-fluoro-2-deoxy-d-glucose (18F-FDG PET CT) with high accuracy and they contribute to decisions regarding diagnosis, staging, recurrence, and treatment response. Here, we report a case of false positive metastatic mediastinal lymph nodes that were diagnosed by 18F-FDG PET CT in a 40-year-old breast cancer patient who had undergone preoperative evaluation. Right paratracheal, prevascular, aorticopulmonary, precarinal, subcarinal, hilar, and subhilar multiple conglomerated mediastinal lymph nodes were revealed in addition to left breast mass and axillary lymph nodes. Mediastinoscopy was performed with biopsy and pathology was reported as granulomatous lymphadenitis. In conclusion, any abnormal FDG accumulation in unusual lymph nodes must be evaluated carefully and confirmed histopathologically.

## 1. Introduction

Breast cancer is the most frequently diagnosed cancer and the leading cause of cancer death among females [1]. It is a major public health problem for women throughout the world. Breast cancer mortality appears to be declining, suggesting a benefit from early detection and more effective treatment [2]. It is accepted that axillary or regional lymph node involvement with metastatic tumor and the presence of distant metastasis are the two most important prognostic factors in patients with breast cancer [3]. Accurate staging is important in determining prognosis and appropriate treatment. Initial breast cancer staging has been based on a multimodality radiological approach; mammography, breast ultrasound, MRI, chest radiography, axillary and abdominal ultrasound, and bone scintigraphy, but this approach is time consuming [4]. Thus, a noninvasive, single-session approach may be desirable. Advanced imaging modalities such as positron emission tomography with computed tomography detects malignancies using 2-[18F]-fluoro-2-deoxy-d-glucose (18F-FDG PET CT) with high accuracy and they contribute to decisions regarding diagnosis, staging, recurrence, and treatment response [6, 7]. The addition of 18F-FDG PET CT in the standard workup of breast cancer may lead to the detection of unexpected metastasis in the initial staging as well as the detection of recurrences [8]. 18F-FDG PET CT has substantial yield in breast cancer patients especially with clinical stage IIB or higher breast cancer [9]. Metastasis to internal mammary or mediastinal lymph nodes is a common occurrence in breast cancer patients with locally advanced or recurrent disease [10]. However, FDG is not a cancer-specific agent, and benign diseases related mainly to infection or inflammation also can show false positive intense FDG uptake, which causes difficulty in differentiating benign disorders from malignant diseases [11]. We report a case of breast cancer patient with intense 18F-FDG uptake in mediastinal lymph nodes related to granulomatous lymphadenitis mimicking metastasis.

## 2. Case Presentation 

**Figure 1:**
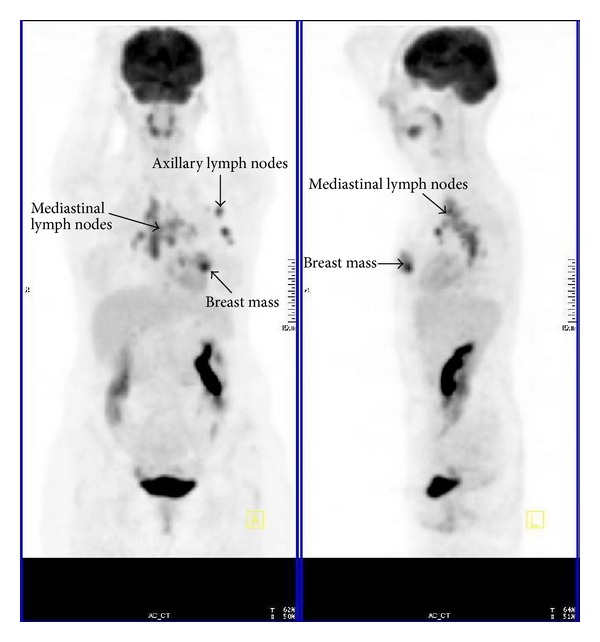
PET in a breast cancer patient shows abnormal FDG uptake.

**Figure 2:**
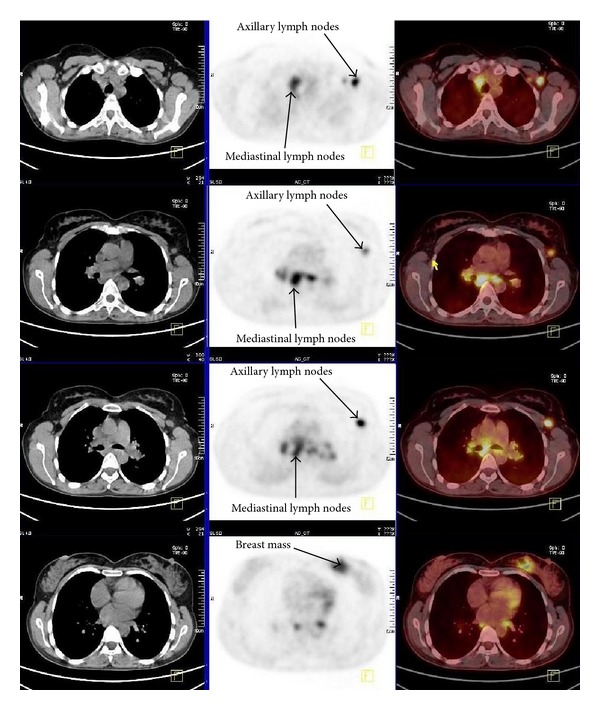
CT (right), PET (middle left), and PET CT (left) transaxial images show intense FDG uptake of mediastinal and axillary lymph nodes and left breast mass.

**Figure 3:**
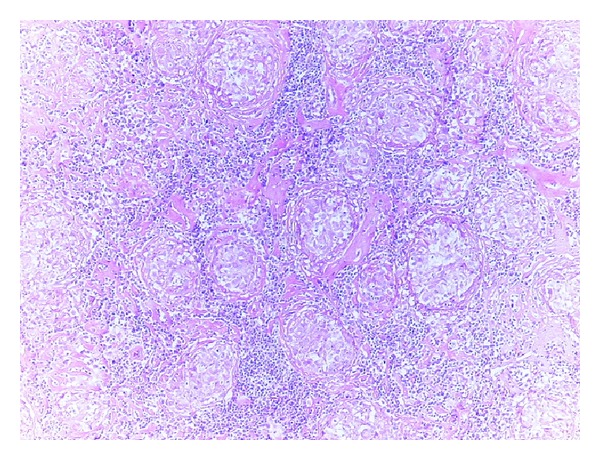
Noncaseating granulomas in lymphoid tissue (H-E, 10x).

**Figure 4:**
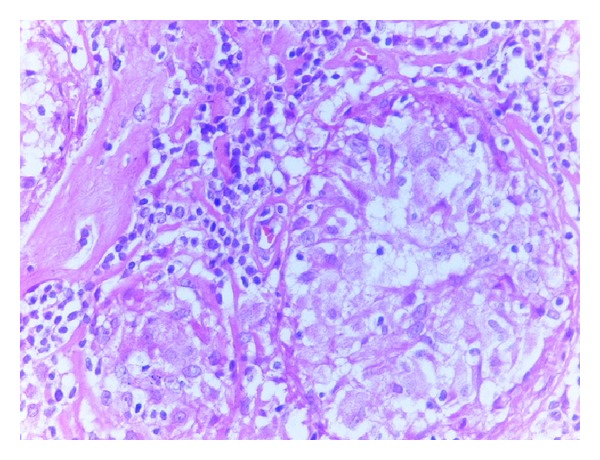
Granulomas showing epithelioid hystiocytes and lymphocytes (H-E, 40x).

A 40-year-old woman presented to doctor with a history of a painless left breast lump without associated nipple discharge. She was otherwise healthy with no other relevant history. Physical examination revealed a nontender, freely movable mass in the left breast. Breast ultrasonography and mammography revealed a 20 × 15 mm periareolar mass with irregular speculated borders without microcalcifications and multiple left axillary hypoechoic malignant lymph nodes with loss of fatty hilum. Fine-needle aspiration cytology was reported as malignant epithelial tumor. She was then referred for staging with 18F-FDG PET CT that was acquired from the base of skull to upper thigh with the arms raised on a Siemens Biograph TruePoint 2008A. CT data was acquired without contrast enhancement and using the following parameters: 130 kV; 60 mAs; pitch 1.5; and slice thickness, 5 mm. The PET CT scan revealed a left breast mass with a size of 27 × 17 mm with a maximum standard uptake value (SUV) of 10,13 and six left axillary lymph nodes measuring up to 17 × 12 mm in size showed intense FDG avidity with SUV value of 8,10 (Figure 1). In addition, right paratracheal, prevascular, aorticopulmonary, precarinal, subcarinal, hilar, and subhilar multiple conglomerated mediastinal lymph nodes with SUV value of 8,16 were revealed (Figure 2). A trucut biopsy was done and reported as invasive carcinoma, estrogen and progesterone receptor status was strongly positive, and HER-2/neu was negative (score 0) with no membrane staining of malignant cells by immunohistochemistry. She was referred to thoracic surgery department, mediastinoscopy was performed under general anaesthesia using a cervical approach, and suspicious lymph nodes were biopsied. Pathology was reported as granulomatous lymphadenitis, auramine-rhodamine (A-R) and Ehrlich-Ziehl-Neelsen (EZN) stainings were negative, no Schaumann or asteroid bodies were observed, and examination of histological sections revealed epithelioid histiocytes, lymphocytes, and a few Langhans-type giant cells with noncaseating granulomas (Figures 3 and 4). Microbiological studies (sputum cultures, tissue staining for acid fast bacilli, and serology) were negative. The patient received 6 cycles of neoadjuvant chemotherapy consisting of taxotere, Adriamycin, and cyclophosphamide (TAC) every 3 weeks. One month after the completion of last cycle of neoadjuvant chemotherapy, PET CT scan revealed right paratracheal, prevascular, aorticopulmonary, precarinal, subcarinal, hilar, and subhilar multiple conglomerated mediastinal lymph nodes with SUV value of 13,13. When compared with the pretreatment PET CT scan, the mass lesion in left breast and left axillary lymph nodes cannot be visualized. The patient was referred for surgery.

## 3. Discussion

PET CT has been recognized as a powerful technique in the detection of malignant tumors, and in the literature several studies have described the usefulness of this imaging for assessing patients with primary breast cancer. It is a noninvasive, all-in-one imaging modality that has been reported to be useful in whole body staging, restaging, and monitoring of treatment response in breast cancer patients [12–14]. Guidelines already suggest that the utility of PET in the staging and management of different tumors including breast cancer, especially to detect unexpected extra-axillary lymph nodes and distant metastases [15]. Results demonstrating the superiority of PET CT over anatomic imaging modalities in the detection of distant metastases are relatively well documented [16]. Fuster et al. found the overall sensitivity and specificity of PET CT in detecting distant metastases to be 100% and 98%, respectively, versus 60% and 83%, respectively, for conventional imaging [8]. Metastasis to extra-axillary lymph nodes is common, but the status of these nodal regions is generally unknown because tissue sampling is not routinely performed in patients with breast cancer [10]. We described a breast cancer patient with mediastinal lymph nodes detected with preoperative PET CT scan. The recognition of disease in extra-axillary lymph nodes may have important implications for the clinical management of patients with breast cancer. For patients suspected of having disease in mediastinal nodal basins this may have important implications with regard to individual patient management, may change local therapy by extending radiation fields, or may change systemic therapy to a more aggressive regimen. However, false positive FDG uptake or false negative PET scans are frequently encountered. Proper interpretation and accurate characterization of an abnormality can be accomplished only if one is aware of possible false positive or negative conditions [11]. Benign conditions causing high uptake of 18F-FDG that have the potential for false positive interpretation in oncologic studies have previously been described [11, 17]. Active granulomatous processes such as tuberculosis, fungal infections and sarcoidosis have been reported to accumulate FDG and can cause false positive results; therefore, acute or chronic infection, or inflammation must always be considered especially in patients with a diagnosis of cancer [11]. High FDG uptake in activated inflammatory cells is due to markedly increased glycolysis and the hexose monophosphate shunt which is stimulated by phagocytosis, with increases of 20–30 times of baseline values [11]. The granulomatous inflammation may be found in the lymph nodes draining the primary tumor either with or without metastatic cancer [5, 18]. The main causes of granulomatous reaction at the drainage sites of malignancies may be idiopathic, foreign body reaction to necrotic tumor or a previous procedure, therapy-related granulomas, and metastasis. In the majority of cases, no definite cause can be found and etiology remains obscure. Some authors suggest the possibility of T-cell-mediated immunological reaction to soluble antigens shed by the tumor which leads to a granulomatous response whereas others attribute it to the persistence of a nondegradable product [19].

In conclusion, if any abnormal FDG accumulation is detected on PET CT scan in unusual lymph nodes, patients with diagnosis of cancer need a thorough preoperative evaluation with histopathological confirmation, thereby allowing the choice of correct staging and curative strategy.
